# Assessing vaginal wall indexes in premenopausal versus postmenopausal women by transrectal linear array high-frequency probe

**DOI:** 10.1186/s40001-023-01378-y

**Published:** 2023-09-28

**Authors:** Mengqi Huang, Yidan Wang, Jiajun Xu, Huiru Xiao, Jingyan Xie

**Affiliations:** 1 Department of Gynecology, Nanjing First Hospital, Nanjing Medical University, Nanjing, 210006 People’s Republic of China; 2 Department of Ultrasonography, Nanjing First Hospital, Nanjing Medical University, Nanjing, 210006 People’s Republic of China; 3 Clinical Academic Department of Global Medical Imaging Products, Shenzhen Mindray Biomedical Electronics Co., Ltd, Shenzhen, 518055 People’s Republic of China

## Abstract

**Objective:**

This study aimed to verify the feasibility of 2D measurement of full-layer thickness of vaginal wall and evaluation of its elasticity by shear wave elastic imaging using transrectal linear array high-frequency ultrasound and to investigate the differences of vaginal wall indexes in premenopausal versus postmenopausal women.

**Method:**

From September to November 2022, a total of 87 women in the Department of Gynecology, Nanjing First Hospital were examined by a sonographer using transrectal linear array high-frequency ultrasound, including 34 women of reproductive age and 53 postmenopausal women. The vagina was divided into upper, middle, and lower segments, and the full-layer thickness of each part was measured. Then shear wave elastography (SWE) was used, and the average value of Young's modulus was used to evaluate the degree of vaginal elasticity.

**Results:**

Transrectal linear array high-frequency ultrasound can clearly display structures of vaginal wall; measurement of the full thickness of the vaginal wall and evaluation of the degree of vaginal elasticity were feasible. There was a statistically significant difference in the thickness of each part of the vaginal wall between pre- and postmenopausal women (*P* < 0.001); there was no significant difference in the vaginal Young's modulus of pre- and postmenopausal women (*P* = 0.073).

**Conclusion:**

Transrectal linear array high-frequency ultrasonography is a non-invasive and feasible method to measure vaginal wall thickness (VWT) and elasticity. There are significant differences in VWT between pre- and postmenopausal women.

## Introductions

With socio-economic development, the progress of population aging worldwide is unstoppable, and the United Nations has clearly set healthy aging as a global goal to solve this problem. The vulvar, vaginal, and lower urinary tract discomfort in menopausal women due to low estrogen status is now collectively known as genitourinary syndrome of menopause, which is difficult to resolve spontaneously without intervention and often presents a slow and aggravating process [1]. Currently, these patients seek medical treatment mainly because of the appearance of urinary and reproductive system symptoms. Previous studies have suggested that the thickness of the vaginal wall is associated with genitourinary syndrome, and we expect to advance the diagnosis of this disease. Because the hormone receptor content in each person's vaginal wall is not consistent, it is necessary to objectively measure VWT to determine the specific judgment. With introduction of transrectal high-frequency transducer in gynecological ultrasound, display of fine structures of pelvic organs can be possible. We used transrectal high-frequency transducer in assessment of vagina, aiming to evaluate vaginal conditions and to obtain significant ultrasound parameters. At present, the diagnosis of GSM diseases lacks of non-invasive and feasible objective basis, but the application of transrectal high-frequency ultrasound makes it possible to evaluate the anatomy of the vagina and provide more information for the comprehensive evaluation of GSM. Therefore, this study discusses the feasibility of measuring the vaginal wall and evaluating the elasticity, so as to provide imaging support for the comprehensive evaluation of GSM in the future.

## Methods

**Fig. 1:**
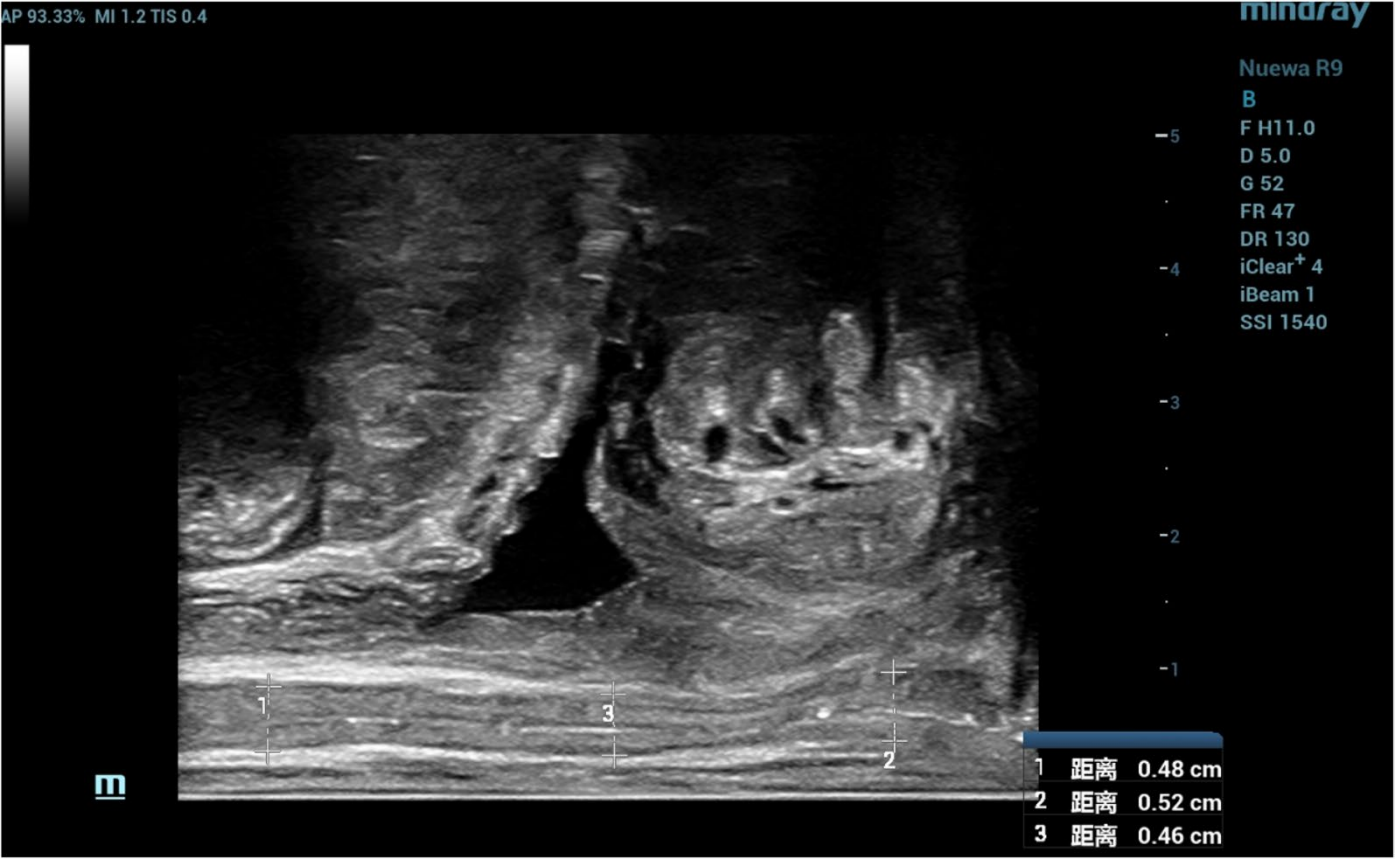
The VWT was measured in its proximal third (1 cm downward from the external cervical orifice), middle third (at the level of the bladder neck), and distal third (1 cm upward from the external vaginal orifice)

**Fig. 2:**
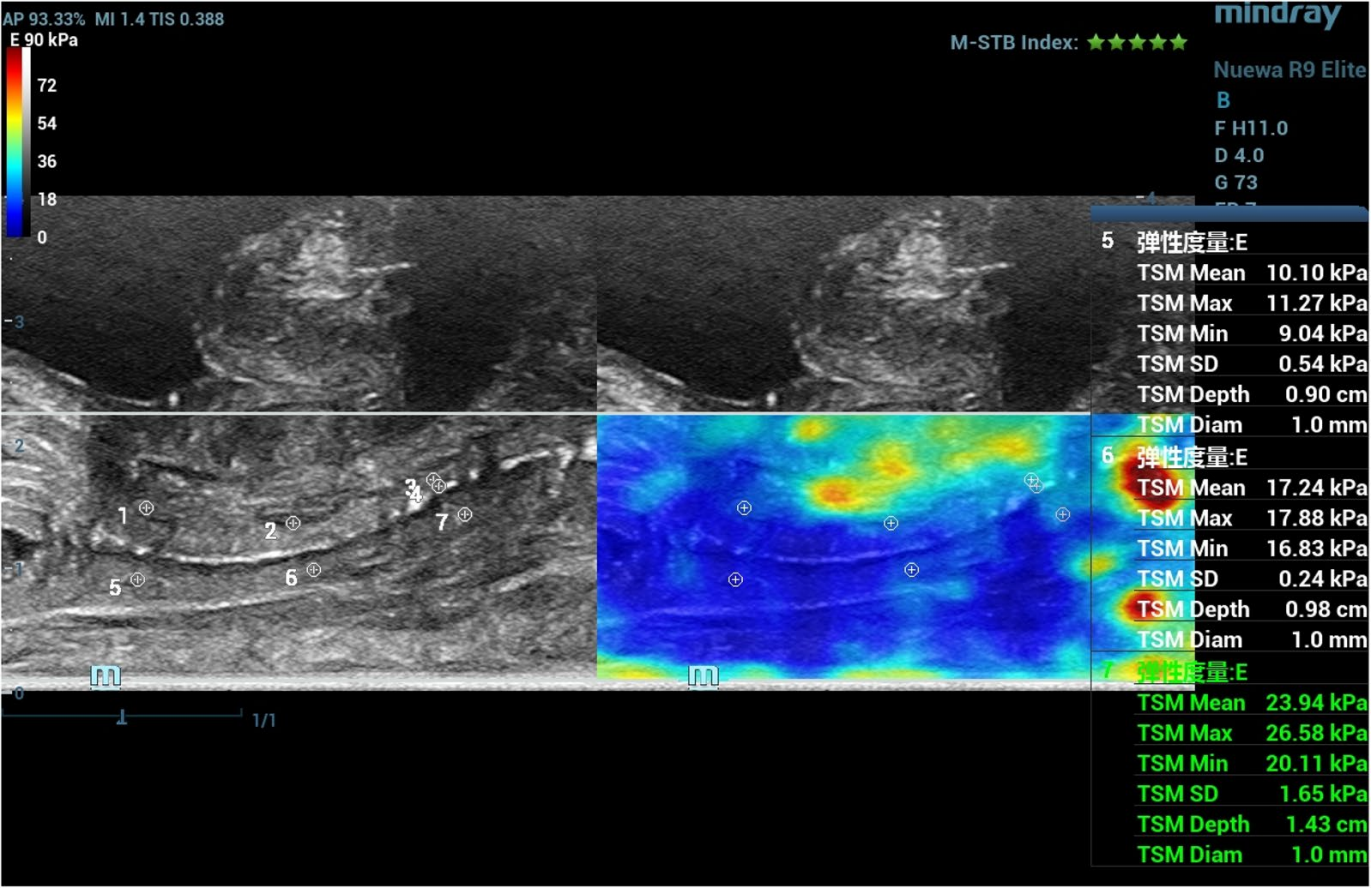
To measure the elastic value of vaginal shear wave, Young's modulus was measured in the anterior, middle, and posterior regions of the anterior and posterior walls of the vagina, respectively, and the average of 6 points was taken as the elastic value of the vagina after the results were obtained

A total of 87 women were selected to undergo pelvic ultrasound in the gynecological ultrasound room of Nanjing First Hospital from September to November 2022. Among them, 34 women of reproductive age and 53 postmenopausal women. Exclusion criteria were as follows: 1. Gynecological surgery: removal of ovaries and/or uterus and/or vagina; 2. Estrogen replacement therapy (including topical/systemic medication) within 3 months; 3. Pelvic organ prolapse [2]; and 4. Pregnancy and delivery within 6 months. This study is descriptive and was approved by the Ethics Committee of Nanjing First Hospital with written informed consent of all participants. In this study, intracavity biplanar high-frequency probe ELC13-4 s (Mindray Nuewa R9 color Doppler ultrasonic diagnostic instrument, linear array probe frequency is 3.2–12.8 MHz, scanning range is 6.48 cm, scanning depth is 1.5-35 cm) was used. 15 min before the examination, the patient was asked to empty the bladder and bowel. During the examination, the bladder lithotomy position was taken, the probe was covered with a protective sleeve and appropriate amount of sterile coupler was taken and then slowly placed in the rectum. The linear array probe is enabled, and the marker point is located in the direction of 12 o 'clock of the patient, so that the probe sound beam is perpendicular to the vaginal wall. When the pubic symphysis, urethra and vaginal air lines were clearly shown in the images, the images were deemed to meet the requirements and measurements were taken. In this study, the VWT was measured at three points in the upper, middle, and lower sections of the vagina anterior and posterior vaginal fornix, at the transition from the proximal urethra and rectum. The measurement points were as follows: the upper part was about 1 cm downward from the external cervical orifice, the middle part was at the level of the bladder neck, and the lower part was 1 cm upward from the external vaginal orifice. The measurement of VWT was performed as parallel between the probe and the vaginal site. Previously, since there was no such examination method, the horizontal vaginal thickness of the bladder triangle was generally measured by abdominal ultrasound. This examination could clearly show the full length of the vagina, so it was possible to choose three points of the vagina to measure the thickness of the vaginal wall. This method reduced the measurement error caused by the pressure of the surrounding tissue on the vaginal wall when measuring the thickness of the vaginal wall at a single point. If the total length of the vagina exceeds the scanning length of the linear array probe, a single section cannot display the full length of the vagina at the same time, the image can be double spelled and measured separately according to the requirements. VWT was defined as the measurement between the outer layer of the anterior vaginal wall and the outer layer of the posterior vaginal wall, by measuring from the limit of the outermost anterior vaginal wall (the fiber tissue of the anterior vaginal wall) to the limit of the outermost posterior vaginal wall (the fiber tissue of the posterior vaginal wall). Three high-echo lines can be observed on this section, and the sound beam from near field to far field was the posterior walls fibers of the vagina, the line of gas between the front and back walls of the vagina and the anterior wall fibers of the vaginal. The distance between the high echo formed by the fiber layer of the anterior vaginal wall and the high echo formed by the fiber layer of the posterior vaginal wall is measured. Measuring Vernier calipers are located on high echo for measurement (Fig. 1). After the measurement of VWT, the SWE was used, and the patient was instructed to hold his breath during the examination. The examiner kept his hand steady, and the dual-image real-time display function was activated. At the same time, the 2D ultrasound image and its corresponding elastic image were observed. The middle regions of the anterior vaginal wall and the anterior, middle, and posterior sections of the vaginal wall were selected as the regions of interest (ROI) with a diameter of 1 cm each time. The Young’s modulus data of six regions were obtained, namely, the anterior wall of the upper vaginal section, the anterior wall of the middle vaginal section, the anterior wall of the lower vaginal section, the posterior wall of the upper vaginal section, the posterior wall of the middle vaginal section, and the posterior wall of the lower vaginal section (Fig. 2). The average Young's modulus (Emean) of the hardness of the vaginal wall was obtained, which provides a quantitative index for the hardness of the vaginal wall. Statistical analysis was performed using SPSS 26.0 statistical software. VWT and Young’s modulus are measurement data conforming to a normal distribution, expressed as mean ± standard deviation. Comparisons between women of childbearing age and postmenopausal women were carried out by independent sample t-test, and *P* < 0.05 was considered statistically significant.

## Results

### Transrectal high-frequency ultrasound imaging of the vaginal ultrasonography with linear array probe

**Fig. 3:**
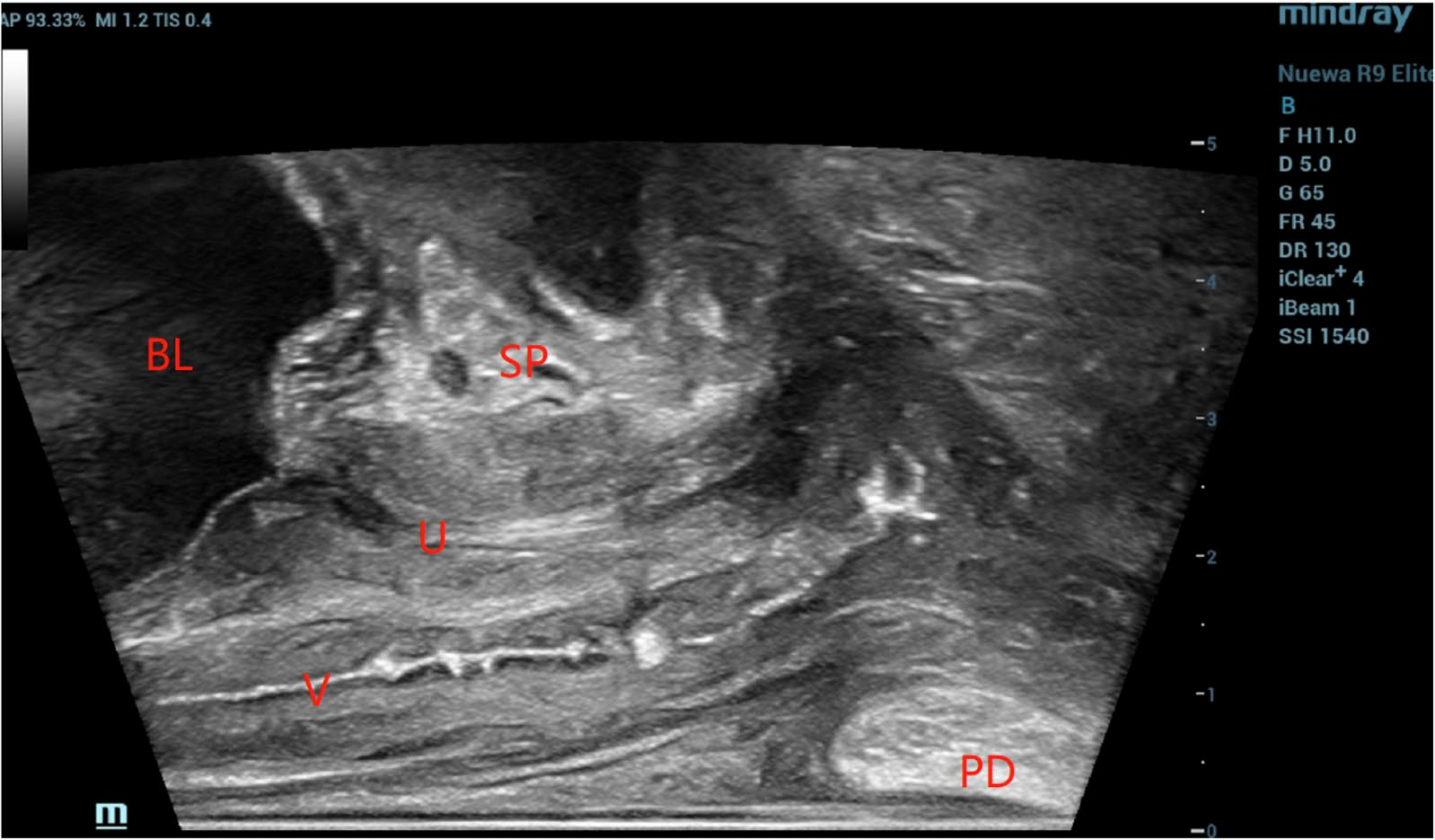
The sagittal surface of the vagina was imaged with linear array probe. The urethra and vagina were clearly visible. *U* urethra, *V* vagina, *SP* pubic union, *BL* bladder, *PD* perineal body

Transrectal high-frequency ultrasound can clearly display the ultrasound images of the vagina and urethra on the sagittal surface (Fig. 3). During this examination, the vaginal wall structure of 2 postmenopausal women was not clearly displayed, and the vaginal wall of women of childbearing age could be clearly displayed. The display rate of vaginal wall structure was 97.7%. During the examination, it was found that the thickness of the horizontal vaginal wall of the bladder neck was the most clear, and the measurement of the upper vaginal wall was located 1 cm below the external opening of the cervix. The cervix of some subjects was distorted, and the probe needed to be rotated to determine the position of the cervix, which increased the difficulty of measurement.

### Comparison of VWT in pre- and postmenopausal women

A total of 87 women were enrolled, including 34 women of childbearing age, aged 26–54 years, and 53 postmenopausal women, aged 52–84 years. The VWT of premenopausal women in the upper, middle, and lower three segments, respectively, were (9.37 ± 2.77) mm, (8.69 ± 2.05) mm, and (9.21 ± 2.24) mm, and that of postmenopausal women were (6.73 ± 2.09) mm, (6.45 ± 1.68) mm, and (6.70 ± 1.98) mm, and there were significant differences in the thickness of the upper, middle, and lower sections of vaginal wall between the two groups (*P* < 0.001). In this study, it was found that no matter for women of reproductive age or postmenopausal women, the vaginal thickness at the level of the bladder neck in the middle of the vagina was the thinnest area in the measurement, but there was no statistically significant difference between the measurement groups of the upper, middle, and lower vaginal segments. Comparison of Young's modulus in pre- and postmenopausal women.

The Young's modulus of the vaginal wall in premenopausal women was (20.81 ± 6.36) KPa, and that of the postmenopausal women was (18.52 ± 5.27) KPa. There was no statistically significant difference in Young's modulus of the vaginal wall in pre- and postmenopausal women (*P* = 0.073) (as shown in Table 1).

**Table 1:** VWT and Young’s modulus of premenopausal versus postmenopausal women

	Premenopausal	Postmenopausal	*P* value
VWT of upper section (mm)	9.37 ± 2.77	6.73 ± 2.09	0.000
VWT of middle section (mm)	8.69 ± 2.05	6.45 ± 1.68	0.000
VWT of lower section (mm)	9.21 ± 2.24	6.70 ± 1.98	0.000
Young’s modulus (Kpa)	20.81 ± 6.36	18.52 ± 5.27	0.073

## Discussion

Estrogen receptors are expressed in the vagina, vulva, pelvic floor muscles, pelvic fascia, urethra, and bladder triangle in women of reproductive age. The ovarian function of postmenopausal women declines, and estrogen levels in the body decrease significantly. The vaginal wall has three layers: from the inside to the outside are mucosal layer, muscle layer and fibrous tissue layer. The mucosal layer contains estrogen receptors and it will change thickness with changes in hormone levels in the female. Because the vaginal mucosa is relatively thin, the ultrasound measurement error is obvious. This study chose to measure the full thickness of the vaginal wall and compare the vaginal state of pre- and postmenopausal women. Women often suffer from menopausal genitourinary syndrome, and most patients present with symptoms and signs of vaginal atrophy [3, 4]. Vaginal atrophy can be quantitatively assessed by measuring the thickness of the vaginal wall, which is also the theoretical basis of this study. Although GSM-related symptoms significantly reduce the quality of life of postmenopausal women, a questionnaire study conducted by Nappi RE et al. in 2011 [5] on menopausal women in seven European and American countries including the UK, the US, and Canada found that 45% of women reported reproductive tract symptoms, but only 4% attributed these symptoms to vulvovaginal atrophy, 63% of women fail to recognize vaginal atrophy as a chronic disease. The findings of this study showed the power of measurements by transrectal high-frequency ultrasound in predicting GSM. Our study showed that the thickness of each part of the vaginal wall measured by transrectal ultrasound had a statistically significant difference in pre- and postmenopausal women, and it was highly likely to be an effective predictor of GSM in future clinical applications. Such ultrasound indicators may even play a role in the quantitative analysis of drug efficacy in the treatment of GSM patients.

In contrast to the routine use of abdominal ultrasound and transvaginal ultrasound in obstetrics and gynecology, high-frequency ultrasound is almost never used to examine the pelvic floor of women, and this probe has traditionally been used to examine prostate and rectal diseases [6]. The linear array high-frequency probe is mainly used in this study, the transducer is located on the side of the probe horizontal plane, which can emit a rectangular acoustic beam. Because the linear array probe is placed in the rectum, the sound beam is completely perpendicular to the vaginal wall in front of the rectum, and the anatomical structure of the vaginal sagittal plane in the near field can be clearly displayed. The limitation of the viewing angle of the end shot probe is overcome. In the case of moderate bladder filling, the length of the vagina and the full thickness of the vaginal wall can be observed and measured. Studies have shown that the length of female vagina is 6–10 cm, and the crystal length of the linear array transmitter used in this study is 6.48 cm. For women with a particularly long vagina, the measurement purpose can be achieved by measuring two images according to the indicating point. In this study, the vaginal wall display rate of 87 women reached 97.7%, so it was feasible to measure the thickness of the vaginal wall with a high-frequency rectal array probe. Two ultrasound images showing the unclear structure of the vaginal wall were reviewed, one woman was obese with intestinal gas and the other woman was constipated. The rectum could not be emptied before examination. It obstructs the transmission of the sound beam from near to far (through the rectal wall to the vaginal wall).

In this study, we found that postmenopausal women had lower total thickness of vaginal wall than women of childbearing age, and there was significant difference in the measurement of the three segments of vaginal wall. Studies with designs similar to our study have found that VWT decreased with menopause. Panayi DC et al. [7] compared the thickness of vaginal wall measured by transvaginal ultrasound with that by histology for the first time and found that the two had good consistency, so 2D ultrasound was considered a reliable method for measuring VWT. Based on this, a high-frequency linear array transrectal probe was designed to measure the VWT. Peker H et al. [8] used a 3D high-frequency intracavity probe to measure the full thickness of the vaginal wall. Whether the above two scholars use 2D or 3D measurement of VWT, because the probe is placed in the vagina, the vagina may be compressed and stretched, thus affecting the accuracy of the measurement. In addition, this method is limited for women with genital tract abnormalities or who are not sexually active. Pereira G et al. [9] injected hydrogel into the vagina to measure the thickness of the front and back walls of the vagina using intravaginal ultrasound, providing a quantitative standard for vaginal relaxation in women. Although the gel was inserted into the vagina to make the vaginal wall more clearly displayed, the sound beam of the vaginal probe was located at the top of the probe, and the vagina could not be completely displayed, and the examination was complicated to create. Balica et al. [10] measured the thickness of vaginal wall by transabdominal ultrasound and found that there were significant differences in the thickness of vaginal wall in women before and after menopause. However, some scholars [11] did not find any correlation between VWT and vaginal mucosa thickness and GSM. During the examination process, the abdominal measurement requires the patient to moderately fill the bladder, and the examination time is unpredictable, and only part of the vagina can be shown due to the influence of the bladder and the pubic branch. In addition, the abdominal probe has a low resolution and cannot clearly distinguish the dividing line between the vaginal wall mucosa, urethra, and rectal mucosa, so the repeatability and accuracy of the measurement results are affected. Therefore, both transabdominal ultrasound and transvaginal ultrasound have shortcomings in measuring VWT, and the transrectal biplanar ultrasound used in this study can solve the above problems. Chinese scholars have pointed out in literature [12] that no matter for postmenopausal women or women of childbearing age, the measurement of VWT by transrectal high-frequency ultrasound has very good repeatability and consistency. In this study, consistent with previous studies, the thickness of vaginal wall in postmenopausal women was thinner than that in women of childbearing age, and the difference was statistically significant. In previous studies, due to the limitation of measurement methods, the thickness of the vaginal wall measured in only one part (that is, the thickness of the vaginal wall at the level of the bladder neck) represents the thickness of the entire vaginal wall, so will the thickness of the vaginal wall in different areas be different? There was no previous literature on the discussion of the thickness of the vaginal wall in different parts, which aroused our interest. Therefore, we designed to measure the thickness of the vaginal wall, respectively, at three representative points of the vagina and found that the thickness of the vaginal wall was not completely consistent. The thickness of the vaginal wall at the level of the bladder neck was relatively thinnest, and the thickness at the upper and lower sections was similar. It is possible that the urethral sphincter at the bladder neck level plays a major role in controlling urine discharge, with greater pressure and greater impact on the vaginal wall. In combination with this study, we suggest that transrectal linear array high-frequency probe measurement of VWT can be used as a non-invasive and quantifiable method for vaginal atrophy, which was the main symptom of GSM. In addition, this study also quantified the compliance of the vaginal wall and compared the values of Young's modulus of GSM and shear wave elastic imaging of women of childbearing age; although the difference was not statistically significant, it also provided a new idea for future research direction.

As a newly developed imaging method, shear wave elastography has been widely used in the diagnosis of breast and thyroid diseases [13]. Compared with traditional strain elastic imaging, shear wave imaging technology detects tissues through the elastic coefficient between tissues and reflects the changes in the internal structure of tissues. Because the way of generating tissue deformation is not affected by the subjective factors of the operator, more accurate and reproducible elastic images can be obtained. Based on the anatomical basis of the vagina, the SWE technique was used in this study to obtain the hardness measurement of the vaginal wall of women of reproductive age and GSM, but no difference was found between the two groups, and it is expected to expand the sample size for further study. Based on the anatomical basis of the vagina, this study used the SWE technique to obtain the hardness measurement of the vaginal wall in women of reproductive age and GSM, but no difference was found between the two. In this study, elastic imaging was performed on the upper, middle, and lower regions of the anterior and posterior vaginal walls, respectively, and the Young's modulus of each region was obtained and then averaged. The average Young's modulus value of the 6 regions represented the hardness of the entire vaginal wall. This method can reduce the fluctuation of Young's modulus data caused by cervical opening or urethra compression of the vagina. The sampling area of elastography ROI could be increased in the later stage. Make it include all vaginas to compare whether there is a difference between the two measurements, and look forward to expanding the sample size for further study.

The transrectal high-frequency ultrasound used in this study was able to show the entire length of the vagina and the full thickness of the vaginal wall without complicated examination preparation. At the same time, the probe is a high-frequency probe, which can clearly show the boundaries of the anterior wall of the vagina, the posterior wall of the vagina, the posterior wall of the urethra, and the anterior wall of the rectum. For perimenopausal and postmenopausal women, transvaginal or transabdominal ultrasound was used to observe the organic changes of the uterus and accessories and assess endometrial thickness, but there was no significant correlation between these indicators and the presence of GSM. Compared with previous experimental explorations, in this study, transrectal high-frequency ultrasound was used to measure VWT, and shear wave imaging was used for vaginal to compare Young's modulus of vaginal wall in postmenopausal women. VWT can reflect the hormone level in the female, and the change of VWT measured by ultrasound can be used as a quantitative analysis for evaluating GSM or symptomatic treatment with medication. In this study, we used a linear array probe in a transrectal biplanar probe to measure the full thickness of the vaginal wall and assess the elasticity of the vaginal wall. The typical symptoms of GSM are associated with vulvovaginal atrophy (VVA), which usually include vaginal dryness, pain, etc and discomfort Symptoms of the urinary system such as estrogen, urinary tract infection and dysuria Lack of associated syndrome. Ultrasound quantifies vaginal atrophy by measuring the thickness of the vaginal wall. The method was feasible, reliable, and economical and convenient. This study showed that there was a statistically significant difference in the total thickness of the vaginal wall pre- and postmenopause, which was also in line with the latest research of Brazilian scholars [10].

The limitation of our study is the fact that it was conducted at a single center. More clinical studies are expected to verify the correlation between vaginal ultrasound indicators and the occurrence and development of GSM. In the later stage, we can combine more abundant ultrasound soft indicators such as urethra length, thickness, and elasticity to do an early screening of menopausal-related diseases. In terms of subject selection, for women who are obese or have intestinal problems such as constipation, this method is limited and the images do not clearly show the structure of the vaginal wall.

## Data Availability

Not applicable.
